# Analysis of Varroa Mite Colony Infestation Level Using New Open Software Based on Deep Learning Techniques

**DOI:** 10.3390/s24123828

**Published:** 2024-06-13

**Authors:** Jose Divasón, Ana Romero, Francisco Javier Martinez-de-Pison, Matías Casalongue, Miguel A. Silvestre, Pilar Santolaria, Jesús L. Yániz

**Affiliations:** 1 Departament of Mathematics and Computer Science, University of La Rioja, 26006 Logroño, Spain; ana.romero@unirioja.es; 2 Department of Mechanical Engineering, University of La Rioja, 26004 Logroño, Spain; fjmartin@unirioja.es; 3 BIOFITER Research Group, Environmental Sciences Institute (IUCA), Department of Animal Production and Food Sciences, University of Zaragoza, 22071 Huesca, Spain; mcasalongue@unizar.es (M.C.); psantola@unizar.es (P.S.); 4 Department of Cell Biology, Functional Biology and Physical Anthropology, University of Valencia, 46100 Burjassot, Spain; miguel.silvestre@uv.es

**Keywords:** Varroa mite detection, deep learning, small object detection

## Abstract

Varroa mites, scientifically identified as *Varroa destructor*, pose a significant threat to beekeeping and cause one of the most destructive diseases affecting honey bee populations. These parasites attach to bees, feeding on their fat tissue, weakening their immune systems, reducing their lifespans, and even causing colony collapse. They also feed during the pre-imaginal stages of the honey bee in brood cells. Given the critical role of honey bees in pollination and the global food supply, controlling Varroa mites is imperative. One of the most common methods used to evaluate the level of Varroa mite infestation in a bee colony is to count all the mites that fall onto sticky boards placed at the bottom of a colony. However, this is usually a manual process that takes a considerable amount of time. This work proposes a deep learning approach for locating and counting Varroa mites using images of the sticky boards taken by smartphone cameras. To this end, a new realistic dataset has been built: it includes images containing numerous artifacts and blurred parts, which makes the task challenging. After testing various architectures (mainly based on two-stage detectors with feature pyramid networks), combination of hyperparameters and some image enhancement techniques, we have obtained a system that achieves a mean average precision (mAP) metric of 0.9073 on the validation set.

## 1. Introduction

Varroa mites, scientifically known as *Varroa destructor*, represent a severe adversary in the world of apiculture and it is one of the most devastating honey bee diseases [1,2]. These parasitic creatures latch onto honey bees and feed primarily on their body fat tissue [3]. Such parasitization has direct consequences for the bees, as it weakens their immune systems, making them more susceptible to diseases and environmental stressors, usually causing a significant reduction in the lifespan and productivity of the affected bees [4,5]. Indeed, *varroosis* stands as the most destructive disease in global beekeeping. Within the European Union, it has become endemic, and is the only beekeeping disease that requires the systematic treatment of bee colonies to ensure parasitization rates remain below hazardous levels. One of the most alarming aspects of Varroa mite infestation is its role as a vector for devastating viruses. The Deformed Wing Virus (DWV), in particular, is a notable pathogen transmitted by Varroa mites [6]. DWV causes physical deformities in infected bees, particularly in their wings, impairing their ability to fly and forage for food. This virus can spread rapidly within a colony, potentially leading to its collapse [7,8].

Given the vital role honey bees play in both pollination and in sustaining the global food supply, it is imperative to prioritize the creation of reliable and efficient techniques for controlling Varroa mites. In fact, the FAO (Food and Agriculture Organizations of the United Nations) estimates that close to 75% of the world’s crops producing fruits and seeds for human consumption depend, at least in part, on pollinators [9]. Thus, control methods are essential not only for the survival of honey bee populations, but also for the crop production that depends on their invaluable pollination services and, finally, for humans.

Efforts to address the infestation of Varroa mites have been ongoing for years, and a variety of treatment methods have been explored [10,11]. These include chemical agents and both biotechnical and biological control [12,13,14]. However, finding a long-term, sustainable solution that effectively manages Varroa mite infestations while minimizing damage to honey bee populations remains a challenging endeavor.

In any case, a crucial part of the process is determining the degree of infestation of a hive. Infestation of a hive is important in the framing of the Integrated Pest Management (IPM) concept and for research purposes. One of the most common methods used to evaluate the level of Varroa mite infestation in a bee colony is to count all the mites that fall onto sticky boards placed on the bottom of a colony [15]. This is a manual process, so a considerable amount of time may be required to estimate the number of mites when the mite loads are high [16]. Varroa mites are barely visible to the naked eye due to their size. They are flat and have a button shape. They are about 1–1.8 mm long and 1.5–2 mm wide, and have eight legs.

This work presents a deep learning approach to locating and counting Varroa mites from images taken with smartphone cameras. In this way, beekeepers could take advantage of this system to perform simple monitoring of the infestation of their hives and take appropriate actions to treat and manage the infestation.

## 2. Related Work

Object detection is a fundamental problem in computer vision that deals with identifying and localizing objects of interest in a digital image. Current state-of-the-art approaches to object detection are based on deep convolutional neural networks (CNN) object detectors. There are two main families of detectors: one-stage methods (such as the YOLO family [17], SSD [18] and EfficientDet [19]) and two-stage methods (the R-CNN family [20,21,22]). In two-stage detectors, one part of the network (the Region Proposal Network, RPN) generates the candidate bounding boxes. Other part of the network analyzes them, ranks their likelihood to be a true positive, and classifies and locates the objects inside. On the other hand, one-stage detectors directly predict the class and location of objects without the need for a separate proposal generation stage. Thus, one-stage detectors are generally faster and more flexible but may sacrifice some accuracy. Two-stage detectors are more accurate, particularly for small objects, but they are slower and require more training data. The Faster R-CNN architecture is a two-stage method for object detection. Its introduction of the Region Proposal Network and the integration of proposal generation and object detection into a single network significantly enhance the overall efficiency and effectiveness of the object detection process. It outperforms previous approaches (like R-CNN [20] and Fast R-CNN [21]) by offering a faster, more accurate, and streamlined approach to object detection [22].

Small Object Detection (SOD) is a more challenging type of object detection, which focuses on detecting small (or even tiny) objects [23]. This task is of great importance for the analysis of satellite images [24] and in biology, since the target objects are usually very small compared to the input image (for instance, the cells in a microscopy image), but is also significant in traffic monitoring [25], drone scenes [26,27], and more [28,29]. It is known that small object detection tasks often present a multitude of additional challenges compared to a standard object detection problem [30].

The low resolution of small objects (compared to the full image) causes *noisy feature problems*, which can hinder neural networks from effectively learning valuable features from their blurred shapes. Additionally, the inherent structure of object detectors, which comprises a backbone with a detection head, tends to introduce some *information loss*: the feature extractor component usually reduces the size of the feature maps and tends to learn high-dimensional features. That is, when an object passes through convolution layers, its size is reduced. This is particularly crucial for small objects, because they are inevitably seen as very few pixels within the network, and their size makes it really difficult to learn from them. Even worse, depending on the network and the input size, they can even disappear after several convolution layers, becoming undetectable.

Various strategies have been explored to mitigate these issues [30,31]. The two most common techniques are to increase image capture resolution and a tiling approach during training and inference. The first technique consists of capturing better-quality images with more information (more pixels). This is not always possible, but it helps to obtain more features of the objects and eventually learn the best information from them [31]. State-of-the-art object detectors allow only images with a fixed size and change the input image size according to this requirement, which usually deforms the small objects in the image and leads to information loss during the resizing process. Meanwhile, the tiling approach simply divides large images into smaller, more manageable sections or tiles. This helps when an image has a higher resolution than the model’s fixed input size because it avoids scaling the image down [32].

Some works have already made use of deep learning models for beekeeping. There are existing papers related to the monitoring of hives [33,34], classification of honey bee comb cells [35], and tracking pollen [36,37], among other applications [38]. However, the use of deep learning for the detection and counting of Varroa mites has not been as widely explored. Some approaches have been used to count Varroa mites directly on adult bees at the hive entrance [39,40]. The authors have recently explored the possibility of using deep learning techniques to detect Varroa mites during the breeding period [23]: hive frames are extracted during such a period; then, Varroa mites and pupae are counted using deep learning techniques. The results were not satisfactory and the neural network exhibited too many prediction failures due to two main reasons: first, the resolution of the images (12Mpx) was not able to capture the features of the the Varroa mites in sufficient detail, meaning that the neural network was unable to learn enough details to distinguish them and therefore confused them with other artifacts such as dust and soil. Second, numerous false positives were detected due to the high similarity between the eyes of the pupae and the Varroa mites themselves. These problems make such an approach unfeasible in practice.

Voudiotis et al. [41] have recently proposed a deep learning beehive monitoring system for detecting Varroa mites. Their approach uses pretrained CNN models and achieves accuracy of close to 70%. Their approach has the disadvantage of requiring an infrastructure in the hives (a camera module component, autonomous device power, a Wi-Fi concentrator, data transmission modules, and so on). Another related work is the one by Bilil et al. [42]: they distinguish between the healthy and the infected bees using the one-stage methods YOLOv5 and a Single-Shot Detector (SSD). Their work is based on an existing dataset [43] containing high-resolution images of bees and Varroa mites; see [42] (Figure 2) for an example. In contrast, our interest lies in using two-stage detectors (specifically, Faster R-CNN) for Varroa mite detection based on the board method and working with pictures that can be taken easily by the beekeeper using a smartphone, in line with the works by Bugnon et al. [44] and Picek et al. [45]. In the first paper [44] the authors also train an object detection model on images showing good results, but unfortunately they do not provide access to the code, neither to the models nor to the dataset. According to their conclusions, they recommend using pictures with a professional camera or the latest generation of smartphones, taking the pictures with good luminosity, without direct sun exposure and using a smartphone holder. The conference paper by Picek et al. [45] proposes the use of two lightweight CNNs, also showing remarkable results. Their approach is different: they begin by extracting the Varroa mite candidates (regions of interest) using classical computer vision techniques (since Varroa mites are characterized by their dark color). They train CNNs with such regions as a classification problem (whether there is a Varroa mite in the image or not). Then, they can predict the infestation level (normal, high risk, critical risk) by counting the number of images that include Varroa mites.

Ultimately, some mobile and cloud applications have arisen that make use of artificial intelligence and image processing techniques to count Varroa mites, such as *beemapping*, *beescanning*, *apisfero* and *Bee Varroa scanner*. However, to the best of our knowledge, either the results are not good or they are commercial apps where the models are not publicly available.

## 3. Materials and Methods

### 3.1. Dataset Description

The dataset consisted of 64 images of dimensions 8064×6048 pixels (48 Mpx) taken under different lighting conditions. The photographs were captured with different smartphone cameras and from the already mentioned sticky boards. The pictures covered an area of approximately 24×17.5 cm. They included green strings to separate the sticky board into frames.

**Figure 1:**
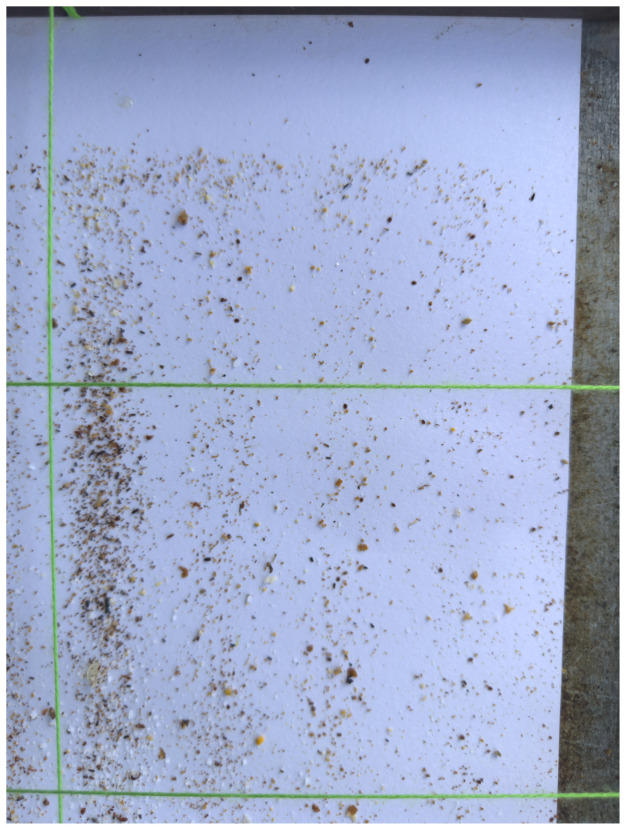
Example of one full picture of the dataset.

**Figure 2:**
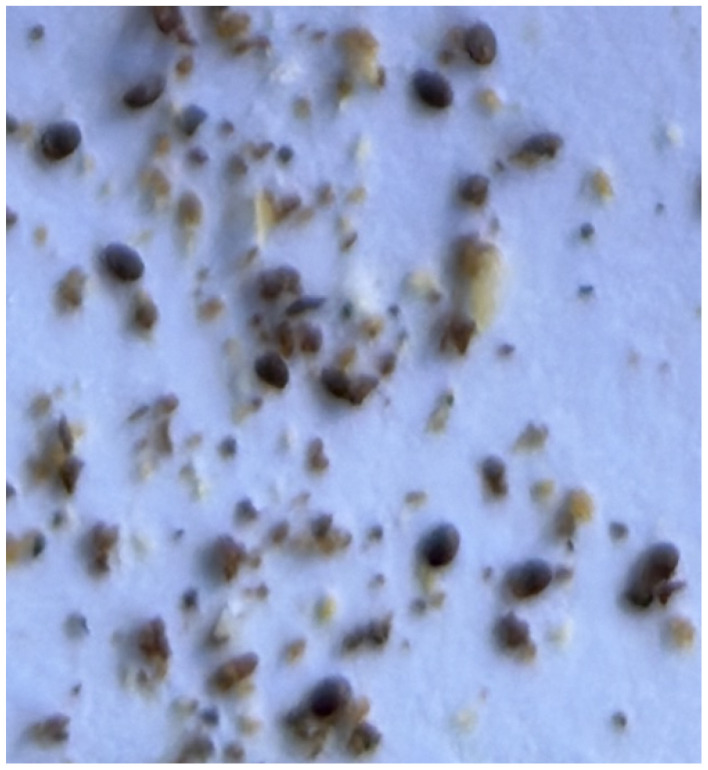
Crop of an image with many artifacts. There are many (blurred) Varroa mites in this part of the image, but also many other elements like dust, soil and dirt.

The images contained Varroa mites, and the number of Varroa mites was highly variable in each image, as shown in Figure 1 (ranging from 1 to 60). The pictures were taken under real conditions and without any preprocessing. The photos also included many other noisy artifacts like dust, dirt, and soil (as can be seen in Figure 2).

**Figure 3:**
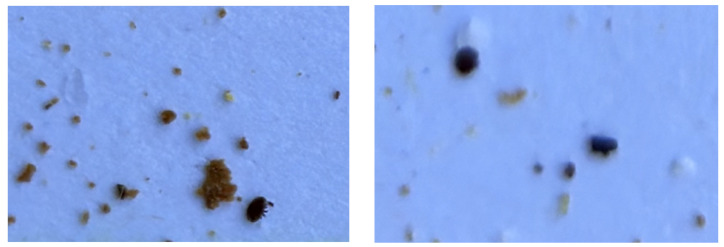
Two crops with different focus. The crops are of identical size and belong to the same image. Both images contain only one Varroa mite, but the image on the right is much more blurred, making identification more difficult (for example, the legs of the Varroa mite are not distinguishable).

Due to the usual limitations of smartphone cameras, some images were not perfectly focused; in fact, the vast majority of photos contained parts that were well focused and parts that were not. See Figure 3 for examples. These realistic conditions are what beekeepers would find in reality when taking a photo with their smartphones, without requiring excessive time to take the picture. Because of this and the tiny size of the Varroa mites, detecting them is a challenge.

The dataset was randomly divided into approximately 75% for training and 25% for validation, while also ensuring that the number of Varroa mites was similar to such percentages in both sets. In total, there were 14 images in the validation set, which contained a total of 227 Varroa mites, whereas 50 images appeared in the training set, containing 580 Varroa mites. The images were manually annotated by experts, who drew a bounding box for each Varroa mite.

### 3.2. Metrics

In order to quantify the performance of deep learning object detection models (i.e., how close the predicted bounding boxes are to the ground truth bounding boxes), a metric must be set. The Intersection over Union (IoU) is a measure of overlap between two bounding boxes, defined as the ratio of the area of overlap between the two bounding boxes to the area of their union. If the IoU between the predicted bounding box and the ground truth bounding box is above a certain threshold, the predicted bounding box is considered a true positive. A perfect match occurs when the IoU is 1, whereas if the bounding boxes are completely distinct then the IoU is 0. The closer to one the IoU is, the better the detection is considered [46].

In object detection problems, the most commonly used metric is the Average Precision (AP), which measures the quality of the detection output by computing the area under the precision-recall curve for a concrete class. Thus, it quantifies how well the model is at identifying objects while considering both precision (the ratio of true positives to all predicted positives) and recall (the ratio of true positives to all actual positives). The calculation of AP relies on the chosen IoU threshold to distinguish between a predicted bounding box being classified as a true positive or a false positive. The mean Average Precision (mAP) is a metric that calculates the average AP across all classes. mAP is typically computed over a range of IoU thresholds, usually denoted in its detailed form as mAP@[0.5,0.95,0.05], which represents the average AP at IoU thresholds from 0.5 to 0.95 in steps of 0.05. Other variants are also widely used in the literature and in object detection benchmarks [47], such as the mAP50, which is the mAP value specifically computed at a constant IoU threshold of 0.5. Thus, the mAP value indicates how good the detector is and also ranges from 0 to 1, where 1 is the optimal result.

Similarly, the mean Average Recall (mAR) is defined as the recall averaged over different IoUs. Pytorch (within its well-known torchmetrics library) includes the concepts mar_1, mar_10 and mar_100, which represent mean average recall for 1, up to 10, and up to 100 detections per image, by default employing the usual range [0.5,0.95,0.05].

This work uses mAP as the main criteria to compare the performance of the models. Specifically, we use mAP50, the mAP computed at a fixed IoU threshold of 0.5. The reason is simple; we are interested in the location of Varroa mites and how many there are, but we do not care that the bounding box is not perfect: we are satisfied with an IoU of 0.50, which is one of the most used threshold values in the literature [47]. The results will also present the mAR score as another measure to evaluate how good each neural network is at detecting all existing Varroa mites. Concretely, we use mar_100 to measure the recall, which again is calculated at a fixed IoU threshold of 0.5. We decided to use mar_100 in order to take into account as many Varroa mites as possible when evaluating the recall and obtain realistic results; because, in fact, most images contain more than 10 Varroa mites.

### 3.3. Workflow for Varroa Mite Detection

This paper presents a deep learning approach to detect and count Varroa mites on sticky board. Since this is a tiny object detection problem with the challenge of possibly having blurred images as input, the proposed approach is composed of several successive steps.

**Figure 4:**
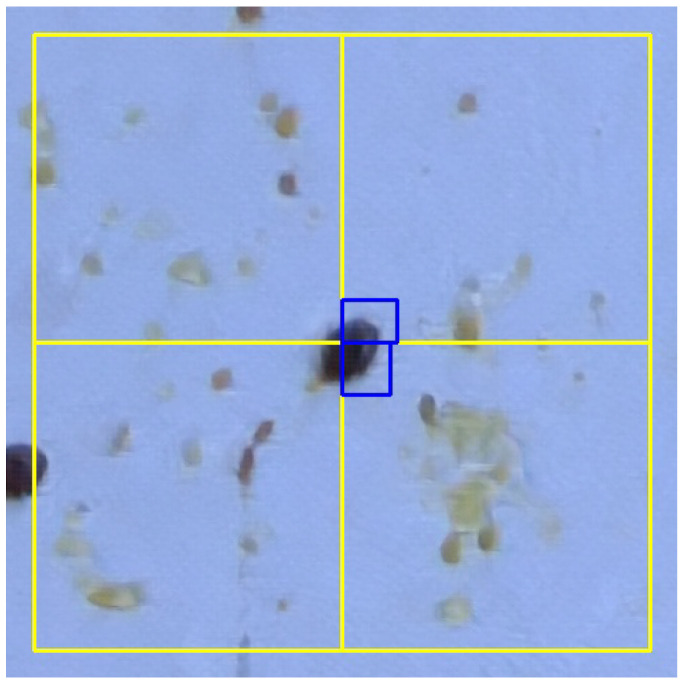
Initial erroneous prediction of a Varroa mite in the union of several tiles. Tiles are drawn in yellow. In blue, two bounding boxes are detected, one for each of the right tiles.

**Figure 5:**
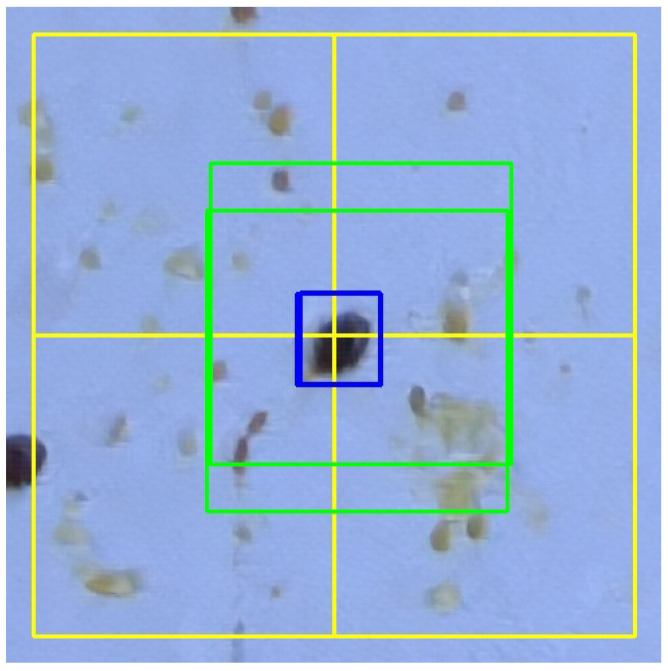
Improved prediction of a Varroa mite in the union of several tiles. Initial tiles are drawn in yellow. For each one of the two bounding boxes in the edges previously detected, a new crop (in green) is considered, which is centered in the middle point of the bounding box. The new predictions produce two similar bounding boxes (in blue), but only the first one is chosen for the final result.

1. Preprocess the input images to improve their quality concretely by motion deblurring using conditional adversarial networks (deblurGAN; see [48,49]). This step takes a blurred image as input and produces the corresponding sharp estimate.
2. Divide each input image (for training and inference) into smaller sections (or tiles). This allows us mitigation of the noisy feature problems and the information loss explained previously. Each image will be processed independently within the neural network.
3. Train a deep learning model based on Faster R-CNN architecture to perform the Varroa mite detection.
4. Finally, after inference, perform an automatic refinement of the predicted bounding boxes. As tiles have also been used during the inference, problems may occur at the unions of two (or more) tiles when combining all the bounding boxes to obtain the final output for the entire image. For instance, if a Varroa mite was precisely placed at the union of several tiles, it may have been detected two or more times (once in each tile; see Figure 4). This step checks, for each tile, if there is a bounding box in any of the edges; if so, a new tile centered on that bounding box is built and the neuronal network performs a new prediction. This new prediction is compared to the initial one and, if the area of the intersection of both bounding boxes is greater than or equal to half of the area of the prediction in the initial tile, the new prediction is chosen instead of the initial one. When several similar predictions are obtained for different tiles, only the first one is added. For instance, in Figure 5, two different new crops (in green) are obtained for the two pieces of Varroa mite detected in the initial tiles. The new bounding boxes (in blue) are very similar and only the first one is considered in the final output of the prediction.

Although each image is divided into a multitude of crops for training and inference, in order to obtain trustworthy results, the chosen metrics (mAP and mAR) are calculated with respect to the complete reconstructed images from these tiles.

### 3.4. Neuronal Network Training

We explored a wide range of neuronal network architectures and hyperparameter combinations to find the highest possible mAP and mAR scores. For this purpose, we carried out multiple experiments adapting Faster R-CNN to different backbones, including the ResNet [50] family (resnet18, resnet50, resnet101 and resnet152), the EfficientNet [51] family (from B0 to B7), and other state-of-the-art architectures, such as the Vision Transformer VITdet [52], ResNext [53] (resnext_101_32x8d) and RegNet [54] (RegNetY_400MF). With many of them, we also experimented with and without including Feature Pyramid Networks (FPN) [55].

For each architecture, we matched the crop size to the corresponding input size of the backbone (for example, we made 224×224 tiles in resnet50 because that was its input size). This step maximized performance, since it prevented the neuronal network from scaling the image down to the backbone input size and, therefore, losing quality. In addition, we ensured that the number of crops with and without Varroa mites was balanced in the training phase, so that the neural networks saw images of both types. For each configuration, we also tested different confidence thresholds values (50 and 90), i.e., a prediction was only considered if the model had a confidence score that was above the threshold. The default pretrained weights of each architecture were used. The maximum number of epochs was set at 600, but we stopped training if there was no improvement within 60 consecutive epochs.

With respect to the training hyperparameters, stochastic gradient descent (SGD) was used to optimize the objective function. The initial learning rate (LR) was set to 0.01, but we used the *ReduceLRonPlateau* technique, which helped models to reach better convergence and avoid being stuck in suboptimal local minima by reducing the LR when there was no enhancement for some prefixed number of epochs [56]. Concretely, this technique was used to reduce LR a factor of 0.75 when the metric did not improve during 10 consecutive epochs.

In addition, we performed image augmentations in each batch to improve the model robustness. Image augmentation is usually very important for object detection tasks, since it increases the effective dataset size, improving generalization and robustness by exposing models to diverse data variations. It prevents over-fitting and mitigates bias. In particular, we use techniques such as rotations, horizontal flip, vertical flip, and modifications on contrast and brightness, which we believe visually introduce the most variety in the data and that can achieve the best performance, taking into account the size and shape of the Varroa mites. The augmentations were applied using their default parameters (the probability of each augmentation was set at 0.50).

### 3.5. Hardware and Software

The experiments were performed on a computer server with two Nvidia RTX 3090 24 GB GPU, two AMD Epyc 7452 2.35 GB CPU and 256 GB RAM. We used the Pytorch library to code the neural networks and perform the training. This library also contains most of the architectures and backbones tested, which were altered by us for our purposes. The image augmentation step was performed using the albumentations library. The dataset is publicly available at https://zenodo.org/doi/10.5281/zenodo.10231844. The code to train the final model can also be found in the repository https://github.com/jodivaso/varroa_detector. This code also allows one to easily modify the hyperparameters (different architectures, different crops and more) to reproduce the results of the experiments performed.

## 4. Results and Discussion

The experiments are summarized in Table 1 and Table 2. Specifically, Table 1 presents the results obtained by the different backbones with 0.50 and 0.90 as confidence thresholds. Table 2 shows a comparison of the best backbones with and without the refinement step and deblurGAN methods.

After testing a multitude of neural network and different architecture combinations, Table 1 shows that backbones with FPNs present generally higher mAP and mAR scores. This is in line with other works, in which it has been shown that FPNs tend to perform better in some small object detection tasks [57,58].

In addition, we have found that the confidence threshold of 0.50 gives better results than 0.90 in the vast majority of architectures tested, providing higher mAP and mAR values. A visual inspection of the prediction scores indicates that scores below 0.90 usually belong to bounding boxes (not necessarily belonging to true Varroa mites) that are cut off at one of the edges of the crop. Therefore, as the detected object is not fully visible in the crop, the neural network does not have all the information and it is not completely clear if it is a true positive or not, resulting in a relatively low confidence score. In our case, according to the results, it makes sense to use 0.50 as confidence threshold to detect all possible Varroa mites (including false positives in some cases) and, in the subsequent refinement step, to adjust the bounding box in order to decide whether it was really a Varroa mite or not.

Overall, the best backbones were resnet18_fpn, resnet50_fpn and resnet152_fpn, obtaining similar mAP and mARs in the validation set. As expected, the results improved after the refinement step was applied (over +0.07 in mAP and +0.1 in mAR; see Table 2). However, it is important to note that resnet_152_fpn is a much more complex model with many more parameters than resnet50_fpn. Similarly, resnet18_fpn is much simpler than resnet50_fpn. Specifically, our Faster R-CNN with resnet18 and FPN has around 11 million parameters, whereas resnet50_fpn has over 41 million and resnet_152_fpn has more than 75 million parameters. Among similar models, it is common practice to choose the simplest one because complex models require more resources with longer training and inference times. In fact, simpler models are known to generalize better, whereas complex and deeper models are prone to over-fitting easily [59]. For this reason, we do not recommend the use of resnet152_fpn in real applications with this dataset, since the execution time is approximately three times that required by resnet18_fpn. In any case, such a model is also available through the repository.

**Figure 6:**
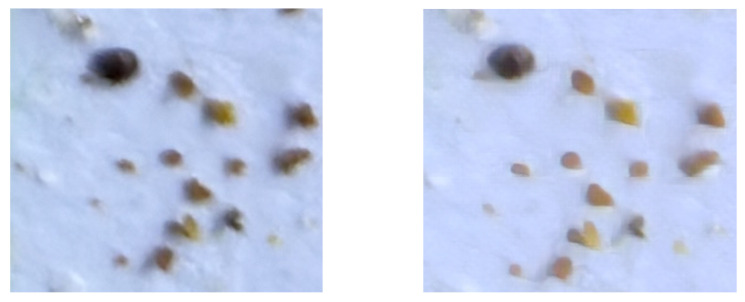
DeblurGAN applied to an image from the dataset. Example taken before and after applying motion deblurring to an image of our dataset. There is one Varroa mite near the upper-left corner. Although, visually, it seems that some image details are simplified, the image is less blurry, which causes metric improvement.

It is important to highlight that both resnet18_fpn and resnet50_fpn improved further (about +0.04 in mAP and +0.01 in mAR) when deblurGAN was applied to the dataset before retraining Faster R-CNN with such backbones, showing that neural networks can sometimes learn to distinguish blurred objects better when advanced techniques, like deblurGAN, are applied. This also shows that it would be possible to use the models without deblurGAN with feasible results, at the cost of a loss of precision. Note that if one applies deblurGAN, but not the refinement step, the metric might not improve in every case. This is because the use of deblurGAN helps neuronal networks to detect more possible candidates that otherwise would have been omitted (they can be false positives, though), but are subsequently screened in the refinement step, and at that point offer an improvement over the version without deblurGAN. An example of deblurGAN applied to an image from the dataset is presented in Figure 6.

**Figure 7:**
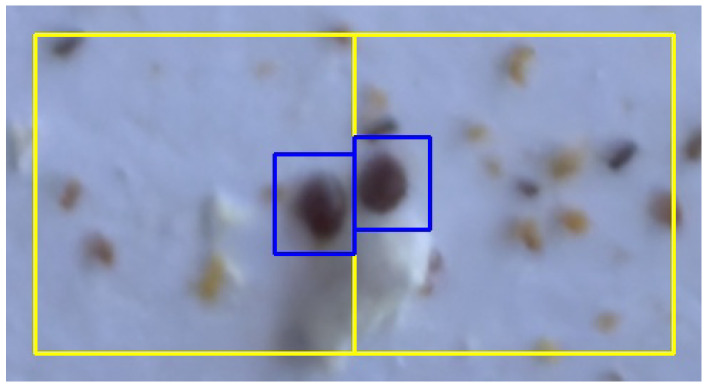
Initial prediction of two Varroa mites at the edge of two consecutive crops. Tiles are drawn in yellow. In blue, two bounding boxes are detected: one for each tile.

**Figure 8:**
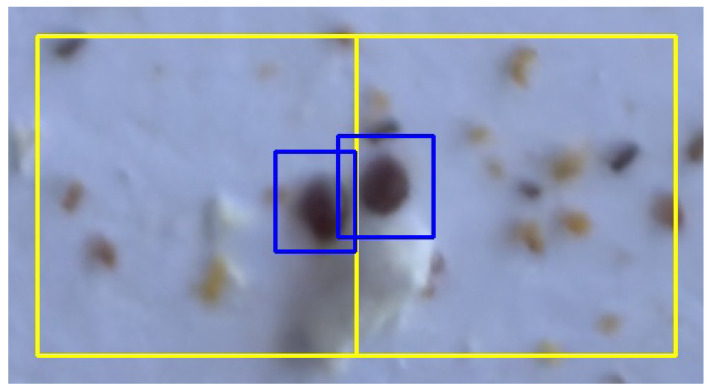
Final prediction of two Varroa mites in the edge of two consecutive crops after the refinement step. Initial tiles are drawn in yellow. The new predictions produce two bounding boxes (in blue), and one of them is bigger than the initial one. The union of the initial bounding boxes would produce an erroneous result.

One could discuss various alternatives for the refinement step. For instance, it is common in the literature to follow an overlap-tile strategy [32,60]. However, this is generally more costly in terms of training time and inference, since the total number of subimages increases. Our approach, as shown, achieves good results and we simply create a new crop for each object detected on an edge. It is also worth noting that some works (including ours) use non-overlapping tiles, where the reconstruction step to obtain the final bounding boxes simply consists of joining pairs of close bounding boxes. This idea, although simple and useful in many cases, is not suitable for our purpose: as the Varroa mites are so small, it may be the case that two different Varroa mites appear to be attached to the same edge in two consecutive crops, which should be derived in two different bounding boxes that should not be joined (see Figure 7 and Figure 8 for an example of this situation, which can be perfectly handled with our approach).

Regarding the number of images in the dataset, it might seem that 64 images are insufficient, since studies using deep learning usually require hundreds or thousands of images [61,62]. However, the total number of Varroa mites in the images is high (there are 807 Varroa mites in the whole dataset) and it is a relatively simple object; thus, the number of images can be considered sufficient for the neural networks to learn to distinguish it (as seen in the results). The pictures were also taken in different lighting conditions and were not an optimal dataset, i.e., they contain blurred parts that are common after capturing them with a smartphone without advanced focusing tools or tripods to increase image stability. This allows the model to be of real applicability for beekeepers. In addition, it should be noted that the total number of images is actually higher, as each image is sliced to the input size of the backbone. For instance, for the ResNet family, for each image the neural network sees 972 sub-images (each image is actually sliced into 972 pieces, 224×224 pixel tiles). Therefore, there are a total of 62,208 images (excluding data augmentation) involved in the training/validation process of the final best neural network.

As for requiring 48 Mpx (or more) for the smartphone cameras, this is because lower resolutions fail to capture enough Varroa mite details and make them indistinguishable with respect to dirt, soil, and other artifacts. Indeed, we performed initial experiments with 9 Mpx and 13 Mpx pictures and they quickly demonstrated the impossibility of such an approach due to the low quality. Nowadays (as of the end of 2023) it is easy to find smartphones produced by different companies that include cameras with high resolution (48 Mpx or more) for a modest price. Therefore, we do not consider this to be a limitation. In addition, the advancement of technology suggests that smartphone cameras are expected to improve in the future and thus, it will be easier for neural networks to detect Varroa mites from their pictures.

The fine-tuning of deep learning models can be an endless task: however, more architectures, more hyperparameters, and more combinations could be tested if unlimited resources were available. In our case, other combinations that have shown good performance in tiny object detection problems could be studied, such as super-resolution techniques (which allow us to artificially increase the resolution of an image) and specific data-augmentation strategies [63]. For instance, some authors copy samples of the small objects and paste them in different positions of the image with one or several random transformations [64,65]. Some of these techniques would probably improve the results of our final model, although we consider that the number of experiments and architectures tested is sufficiently large and the results are already good enough.

Regarding the resources needed to perform the inference using the current models, it is totally unfeasible to run them on a smartphone directly: the images have to be sent via the internet to a computer with a GPU, and the result is returned by the computer. This is due to two reasons: first, two-stage large neural networks (such as Faster R-CNN) have been used to detect enough details of the Varroa mites to distinguish them. With simpler neural networks, the results would be much poorer. Second, for each image, hundreds of subimages must be analyzed, so it would not be possible to make an inference on a smartphone in a moderate time. The inference time is very dependent on the available GPU, but with an Nvidia RTX 3090 (like the ones used for training), it takes a few seconds per complete image (concretely, it takes about 15 s using resnet18_fpn as backbone and about 22 s using resnet50_fpn), which includes the refinement process of the bounding boxes. If a more modern GPU (e.g., a consumer-grade one such as the Nvidia RTX 4090 or datacenter-grade one such as the Nvidia L40) was used, this time would be lower. Moreover, this inference time could also be reduced using a GPU farm (i.e., several GPUs), since the task is highly parallelizable (each subimage can be analyzed on different GPUs independently). It is important to stress that the training of the deep learning models has been performed on a powerful server for efficiency reasons: each training phase usually takes about a day, and the server has allowed us to perform in parallel the dozens of experiments performed with the different combinations of architectures and hyperparameters in an agile way. However, in order to perform inference with the best model obtained, is not necessary to own a 24 GB VRAM GPU such as the ones we used in the training process, since running predictions on a neural network is not as taxing as training a neural network model. A more modest 4 GB GPU would be enough. All this need for computational power is also a consequence of having a realistic and, at the same time, complicated dataset that includes blurred pictures with many artifacts and waste.

It is also worth mentioning that beekeepers do not need to know the exact number of Varroa mites that appear on the sticky board; in fact, an estimation is enough to determine the degree of infestation of the colony, and therefore a small detection error can be assumed. Because of this, we do not consider it necessary to apply model ensemble techniques that could improve the mAP score of the models slightly, with the additional cost of a significant increase in the execution time.

The developed models can be directly reused to detect Varroa mites with the sticky board method following our approach, or they can also be used as a basis for either transfer learning or semi-supervised learning on another Varroa mite dataset under different conditions, i.e., the models can be used to automatically annotate other images, subsequently manually checking the labels.

All the code and models are publicly available. The developed model can also be used in Google Colaboratory. Our idea is to develop free software to detect Varroa mite, and this work is the first step towards this goal.

Finally, it is important to note that the dataset images contain strings that separate the sticky board into frames, which are used as a guide when capturing the photo. The developed models detect Varroa mites in the whole image. However, if one is only interested in counting inside the frames and not in the whole picture, this is also possible: this step can be performed manually by simply marking four points (the boundary corners of the area to count), or it is very simple to detect this area automatically using the Hough transform, which is a well-known method for detecting straight lines in an image.

## 5. Conclusions

This paper has presented some deep learning models that allow Varroa mite detection with good precision and recall using simple smartphone cameras and consumer-grade GPUs. Therefore, we believe that it is feasible that beekeepers (in particular, for instance, through services provided by each beekeeper association) can use these techniques to monitor and control Varroa mite infestation. Our code, the models, and the dataset are publicly available and can be freely reused and adapted to each context.

## Figures and Tables

**Table 1:** Summary of the results of the experiments with different backbones and confidence thresholds.

Backbone	Confidence Threshold	mAP	mAR	Epochs
resnet18	0.90	0.662	0.714	210
resnet18	0.50	0.719	0.814	180
resnet34	0.90	0.727	0.788	166
resnet34	0.50	0.736	0.810	180
resnet50	0.90	0.657	0.699	294
resnet50	0.50	0.693	0.799	154
resnet101	0.90	0.710	0.803	505
resnet101	0.50	0.663	0.807	322
resnet152	0.90	0.718	0.784	425
resnet152	0.50	0.725	0.822	431
vitdet	0.90	0.579	0.747	204
vitdet	0.50	0.579	0.762	226
efficientnetb0	0.90	0.692	0.777	496
efficientnetb0	0.50	0.735	0.836	576
efficientnetb1	0.90	0.532	0.658	443
efficientnetb1	0.50	0.697	0.792	432
efficientnetb2	0.90	0.623	0.691	382
efficientnetb2	0.50	0.731	0.844	364
efficientnetb3	0.90	0.551	0.587	389
efficientnetb3	0.50	0.744	0.844	500
efficientnetb4	0.90	0.548	0.654	437
efficientnetb4	0.50	0.647	0.799	481
efficientnetb5	0.90	0.655	0.773	392
efficientnetb5	0.50	0.453	0.498	398
efficientnetb6	0.90	0.476	0.517	408
efficientnetb6	0.50	0.708	0.799	412
efficientnetb7	0.90	0.486	0.520	375
efficientnetb7	0.50	0.641	0.744	365
resnext101_32x8d	0.90	0.677	0.796	437
resnext101_32x8d	0.50	0.683	0.788	163
resnext_101_32x8d_fpn	0.50	0.769	0.844	148
resnext_101_32x8d_fpn	0.90	0.774	0.833	165
regnet_y_400mf	0.90	0.633	0.714	294
regnet_y_400mf	0.50	0.703	0.784	208
resnet18_fpn	0.90	0.751	0.833	162
resnet18_fpn	0.50	**0.780**	0.848	162
resnet34_fpn	0.90	0.711	0.807	83
resnet34_fpn	0.50	0.648	0.814	97
resnet50_fpn	0.90	0.754	0.825	191
resnet50_fpn	0.50	0.777	0.844	158
resnet101_fpn	0.90	0.746	0.836	180
resnet101_fpn	0.50	0.754	0.844	145
resnet152_fpn	0.90	0.776	0.848	153
resnet152_fpn	0.50	0.779	**0.851**	201

**Table 2:** Results of the best backbones with and without applying deblurGAN and the refinement step.

Backbone	Confidence Threshold	Refinement Step	deblurGAN	mAP	mAR
resnet18_fpn	0.50	No	No	0.780	0.847
resnet18_fpn	0.50	Yes	No	0.849	0.948
resnet18_fpn	0.50	No	Yes	0.775	0.851
resnet18_fpn	0.50	Yes	Yes	0.883	0.955
resnet50_fpn	0.50	No	No	0.777	0.844
resnet50_fpn	0.50	Yes	No	0.858	0.963
resnet50_fpn	0.50	No	Yes	0.780	0.844
resnet50_fpn	0.50	Yes	Yes	**0.907**	**0.967**
resnet152_fpn	0.50	No	No	0.779	0.851
resnet152_fpn	0.50	Yes	No	0.894	0.963
resnet152_fpn	0.50	No	Yes	0.709	0.807
resnet152_fpn	0.50	Yes	Yes	0.851	0.941

## Data Availability

The dataset and the best model are publicly available at https://zenodo.org/doi/10.5281/zenodo.10231844. The code to perform the experiments and train the final model can also be found in the repository https://github.com/jodivaso/varroa_detector.
